# Abstract of Paper on Abortion

**Published:** 1897-04

**Authors:** R. J. MacGuire

**Affiliations:** Concord, N. H.


### ABSTRACT OF PAPER ON ABORTION.[1]

By R. J. MacGuire, V.S.,
CONCORD, N. H.

First referring to the disposition of most cattle-owners to ignore veterinary assistance, which has contributed to its extension, perpetuation, and great losses, and passing rapidly over the accidental traumatic causes, he dealt more particularly with the form recognized as infectious and the period in the mare most often noted—the 120th to the 270th day; in the cow from the 150th to the 210th day. As to its infectious character he quoted from the researches of the Royal Agricultural Society of England, which recognized a specific cause for the trouble; also to Denmark's investigation of this disease through the veterinary profession and their conclusions as to its infectious character, noting the conclusions of Ex-State Veterinarian Turner, of Missouri, as to its micro-organismal origin. His own conclusions were given as to bulls being the most potent method of transmission through their covering cows which had aborted, and then transmitting it to others through service of other animals; the absence of premonitory symptoms in the cow and the only noticeable ones of slight uneasiness, stamping, and switching of tail in mares. As to treatment, the wisdom of local dis-

[1] Read before the New Hampshire Veterinary Medical Association, October 8, 1896.

infection through destruction of the young fœtal membranes and local cleansing of the parts daily of the aborting animals with a 1 per cent. solution of carbolic acid, a 1 to 1000 parts solution of corrosive sublimate, or a 2 per cent. solution of creolin; separation of the sick from the well, though he thought this was of little real value on the average farm, owing to the lack of proper accommodations for carrying it out. He urged suitable legislation looking to its control.

